# Serum uric acid: creatinine ratio (UCR) is associated with recurrence of atrial fibrillation after catheter ablation

**DOI:** 10.3389/fendo.2023.1110102

**Published:** 2023-05-19

**Authors:** Yujiao Zhang, Yanxin Wang, Xuesong Yang, Zhan Li, Luxiang Shang, Yinglong Hou

**Affiliations:** ^1^ Department of Cardiology, Shandong First Medical University, The First Affiliated Hospital of Shandong First Medical University & Shandong Provincial Qianfoshan Hospital, Shandong Medicine and Health Key Laboratory of Cardiac Electrophysiology and Arrhythmia, Jinan, China; ^2^ Department of Cardiology, Shandong Provincial Qianfoshan Hospital, Shandong Medicine and Health Key Laboratory of Cardiac Electrophysiology and Arrhythmia, Cheeloo College of Medicine, Shandong University, Jinan, China

**Keywords:** atrial fibrillation, catheter ablation, recurrence, uric acid: creatinine ratio (UCR), arrhythmia

## Abstract

**Background and aims:**

Studies showed that elevated preoperative serum uric acid(SUA) levels are associated with recurrence of atrial fibrillation(AF) after catheter ablation. UA:creatinine ratio(UCR - UA normalised for renal function) has appeared as a new biomarker and is considered to reflect endogenous UA levels preferably because it eliminates the influence of renal function. This study aimed to investigate the correlation between UCR and recurrence of AF after catheter ablation.

**Methods and results:**

A total of 233 consecutive patients with symptomatic, drug-refractory AF underwent catheter ablation. All participants underwent history-taking, physical examination and blood biochemistry analysis at baseline. After a mean follow-up of 23.99 ± 0.76 months, recurrence ratios for each UCR quartile (from lowest quartile to highest) were 10.9%, 23.6%, 23.6%, and 41.8%, respectively (P = 0.005). Multivariate Cox regression analysis revealed that UCR was an independent predictor of AF recurrence (HR 1.217, 95%CI 1.008-1.468; P = 0.041). Subgroup analysis showed that UCR was associated with AF recurrence in paroxysmal AF (HR 1.426, 95% CI 1.092-1.8608; P = 0.009) and in male patients (HR 1.407, 95% CI 1.015-1.950; P = 0.04). A cut-off point of 4.475 for the UCR had sensitivity of 65.5% and specificity of 59.6% in predicting AF recurrence (P = 0.001).

**Conclusion:**

Our results demonstrate that elevated preoperative UCR is associated with recurrence of AF after catheter ablation, and it indicate UCR maybe a predictive factor for the recurrence of AF.

## 1 Introduction

Atrial fibrillation (AF), the most common sustained cardiac arrhythmia, is associated with an increased long-term risk of stroke, heart failure, and all-cause mortality (1). Over the past 20 years, catheter ablation has become an increasingly popular means of procuring rhythm control for patients with symptomatic and drug-refractory AF. However, there is still a risk of recurrence after ablation, occurring in approximately 25-50% of patients (2). It is important to explore the risk factors related to recurrence of AF and carry out intervention to prevent recurrence.

At present, the risk factors known to be associated with recurrence of AF include hypertension, coronary heart disease, obesity, obstructive sleep apnoea (OSA), and other inflammatory or metabolic diseases. However, there is limited evidence to support the view that these serum biomarkers could be used to detect pathogenesis of AF recurrence. Serum uric acid (SUA), an important indicator of metabolism, has been associated with recurrence of AF (3). Nevertheless, renal function is also an influential factor for AF. Given the fact that renal clearance of SUA is often impaired during kidney injury, renal function is the major confounder in studies for the association between serum UA levels and CVD (4, 5).

A study suggested that serum UA to creatinine (Cr) ratio (UA : Cr, UCR) might be a better predictor excluding factors of kidney injury than serum UA alone. Higher serum UCR levels correlated with an increased risk of all metabolic syndrome components (4). Recently, the components of metabolic syndrome were also found to be associated with high serum Cr levels. Notably, the subjects with higher levels of serum UCR have more cardiometabolic risk factors and hence the serum UCR may be useful in determining prognosis for metabolic syndrome. In addition, previous studies have shown that this biomarker was closely related to metabolic syndrome, renal disease progression, as well as total and cause specific mortality (6–8). However, its relationship with AF recurrence still required investigation. Therefore, we tried to generate a new index using renal function-normalised UA and tested whether it is superior to UA as the predictor of AF recurrence after catheter ablation.

## 2 Methods

### 2.1 Study design and population

For this retrospective study, we included consecutive Chinese patients with drug-refractory AF who had undergone radiofrequency (RF) catheter ablation for the first time between January 2018 and May 2021 at Department of Cardiology, the first affiliated hospital of Shandong First Medical University. According to the guidelines for the diagnosis and management of AF, a standard 12-lead ECG recording or a single-lead ECG tracing of ≥ 30 s showing heart rhythm with no discernible P waves and irregular RR intervals (when atrioventricular conduction is not impaired) is diagnostic of clinical AF (9). The exclusion criteria were as follows: (i) left ventricular ejection fraction (LVEF) < 50% and left atrium (LA) diameter > 55 mm; (ii) estimated Glomerular Filtration Rate (eGFR) < 15 mL/min. Written informed consent had been obtained before participation and the study was approved by the institutional ethical review committee. Prior to the procedure, informed consent was obtained from all patients, in accordance with our hospital guidelines. The study was approved by the Ethics Committee of the First Affiliated Hospital of Shandong First Medical University.

### 2.2 Baseline data collection

Detailed medical histories related to cardiovascular and systemic conditions of all the patients were collected. Baseline characteristics, including age, sex, height, weight, smoking history, alcohol consumption, and drug history, were assessed. Complications related to cardiovascular disease, including diabetes, hypertension, hyperlipidaemia and coronary atherosclerotic heart disease were evaluated. The CHA2DS2-VASc scores were calculated for each patient according to 2020 ESC Guidelines for the diagnosis and management of AF (9). Fasting blood samples were collected from all participants before catheter ablation. Hematological indicators were measured using standard laboratory procedures. SUA, creatinine, triglyceride (TG) and superoxide dismutase (SOD) concentration were measured by the colorimetric method. Total cholesterol (TC), high-density lipoprotein cholesterol (HDL-C), low-density lipoprotein cholesterol (LDL-C) and glycated hemoglobin A1c (HbA1c) were measured by enzyme colorimetry, the direct method of the catalase clear method, selective elimination and high efficiency liquid chromatography, respectively.

### 2.3 Preoperative preparation

All patients underwent transthoracic echocardiography and transoesophageal echocardiography to examine left atrium diameter (LAD), LVEF, valve parameters and to define no left atrial thrombus within 48 hours before the procedure. Pulmonary vein (PV) CT was used to assess the structure of the PVs. Novel oral anticoagulants were continued until 12h before the procedure. Vitamin K antagonist (VKA) was stopped 2 days before the intervention to achieve an international normalised ratio between 1.5 and 2.0, and subcutaneous low-molecular weight heparin twice per day was administered as bridge therapy. Antiarrhythmic drugs except amiodarone were stopped five half-lives before the procedure, and restarted on the following day.

### 2.4 Ablation procedure

Procedures were performed under modest sedation with fentanyl. The dose of heparin used was 70-100U/kg body weight. The left atrial structure was reconstructed under the guidance of three-dimensional mapping system (CARTO-3, Biosense Webster Inc., Irvine, CA, USA). Using trans-septal access, the Lasso or PentaRay mapping catheter was placed sequentially within each PV to record baseline PV potentials. Circumferential PV isolation was performed using an irrigated-tip contact-force sensing RF ablation catheter (Thermocool SmartTouch, Biosense Webster) for patients undergoing radiofrequency ablation. Point by point ablation along the PV vestibule (power mode, 30-40 W, maximum 43°C, infusion rate 15 mL/min) was performed. The contact force applied prior to lesion delivery was 20 g (acceptable range 10-30g), with a minimum individual target lesion duration of 400 gram-seconds force-time integral. The ablation points were connected into a ring line around the left and right PVs, and complete electrical isolation of the PVs was verified. Bidirectional conduction block from the atrium to the PVs was judged as the successful ablation endpoint identified by a mapping catheter (10). Electrical cardioversion was used to restore sinus rhythm when necessary.

### 2.5 Post-procedural follow-up

Antiarrhythmic and oral anticoagulant drugs were continued for 3 months if there was no recurrence of arrhythmia. All patients were followed up with continuous electrocardiogram monitoring for 24 h before discharge. A 12-lead electrocardiogram (ECG) and 24-hour Holter recording were undertaken before discharge, at three months after the ablation procedure, and twice every year subsequently. In addition, telephone interviews were conducted by a referring physician every 6 months. If a patient became symptomatic, a new ECG or 24-hour Holter recording was performed. Recurrence was defined as an episode of AF, atrial flutter or atrial tachycardia of at least 30 seconds duration confirmed by ECG or Holter recording more than 3 months after the AF ablation. The follow-up time was at least 6 months.

### 2.6 Statistical analysis

Continuous data were presented as means ± standard deviation and compared using Student’s t-test. Categorical data were presented as percentages of the total in each category and were compared using the chi-squared test. Participants were stratified by serum UCR quartiles. Cox proportional hazard regression analysis was used to test the effect of the variables on AF recurrence, adjusted for other variables. Kaplan–Meier analysis was used to analyse time to recurrence of AF after ablation. The risk was presented as hazard ratio (HR) at 95% confidence interval (95% CI). Correlations were assessed using Spearman’s correlation coefficient. Receiver operating characteristic (ROC) curve analysis was used to determine the predictive value of UCR and AF subtype for incident AF recurrence. All tests were two-sided, and P-values < 0.05 were considered statistically significant. Data analysis was performed using SPSS software, version 24.0 (SPSS, Inc.).

## 3 Results

### 3.1 Patient baseline clinical characteristics

The study population consisted of 233 consecutive patients (mean age 61.44 ± 9.43 years, 128 males) with either paroxysmal (n = 171) or persistent (n = 62) AF according to the exclusion criteria. The mean SUA and Cr of this cohort were 310.1 ± 80.35 mmol/L and 71.94 ± 16.01 mmol/L, respectively. PV isolation was achieved in all patients. After a mean follow-up of 20.74 ± 12.01months, 55 (23.60%) patients had AF recurrence. The mean UCR values had a higher level of AF recurrence than AF non-recurrence (4.868 ± 1.191 vs 4.291 ± 1.246, P = 0.003). Hypertension and diabetes were present in 27/55 (49.09%) and 7/55 (12.73%) of individuals with recurrence, 88/178 (49.43%) and 32/178 (17.98%) of non-recurrence patients, respectively. Patients with AF recurrence had a greater prevalence of persistent AF (PeAF) (41.82% vs 21.91%, P < 0.01) than those without AF recurrence. Baseline characteristics and demographic features of the study population are given in **Table 1**.

**Table 1:** Baseline characteristics of the study population(n=233).

	Total (n=233)	Recurrence (n=55)	No recurrence (n=178)	P
Clinical data				
Age(years)	61.44 ± 9.43	60.56 ± 8.92	61.71 ± 9.60	P=0.423
Gender, male; n (%)	128(54.9%)	26(47.3%)	102(57.3%)	P=0.191
BMI	26.01 ± 3.54	25.87 ± 3.42	26.05 ± 3.58	P=0.786
Smoking, n (%)	66(28.3%)	11(20.0%)	55(30.9%)	P=0.117
Alcohol, n (%)	66(22.75%)	11(20.0%)	42(23.60%)	P=0.578
Hypertension, n (%)	115/233(49.36%)	27/55(49.09%)	88/178(49.43%)	P=0.964
Diabetes mellitus, n (%)	39/233(16.74%)	7/55(12.73%)	32/178(17.98%)	P=0.362
CAD, n (%)	82/233(35.19%)	20(36.36%)	62(34.83%)	P=0.835
CHA2DS2-vasc Score	2.318 ± 1.641	2.33 ± 1.846	2.32 ± 1.577	P=0.96
Echocardiographic parameters				
LA, mm	38.83 ± 5.26	39.21 ± 5.69	38.71 ± 5.13	P=0.553
LVEF, %	63.45 ± 5.65	64.0 ± 5.94	63.28 ± 5.56	P=0.423
Laboratory parameters				
D-dimer, mg/L	0.360 ± 0.45	0.288 ± 0.47	0.381 ± 0.44	P=0.195
WBC, ×10^9	6.04 ± 1.56	5.98 ± 1.41	6.06 ± 1.61	P=0.731
Neutrophil,×10^9	3.56 ± 1.34	3.38 ± 1.05	3.62 ± 1.41	P=0.261
TG, mmol/L	1.33 ± 0.72	1.50 ± 0.80	1.28 ± 0.69	P=0.058
TC, mmol/L	4.05 ± 0.95	4.05 ± 0.91	4.05 ± 0.97	P=0.998
LDL-C, mmol/L	2.31 ± 0.73	2.32 ± 0.67	2.31 ± 0.75	P=0.913
HbA1c, %	6.116 ± 0.795	6.270 ± 0.932	6.041 ± 0.709	P=0.067
BNP, pg/mL	147.7 ± 185.1	149.9 ± 198.1	147.0 ± 178.6	P=0.922
creatinine, mmol/L	71.94 ± 16.01	68.80 ± 13.79	72.92 ± 16.56	P=0.096
UA, mmol/L	310.1 ± 80.35	328.8 ± 78.71	304.3 ± 80.19	P=0.047
SOD, U/mL	162.2 ± 20.44	165.6 ± 25.82	161.2 ± 18.43	P=0.18
UA : SOD	1.914 ± 0.543	1.93 ± 0.508	1.908 ± 0.555	P-0.803
UCR	4.427 ± 1.255	4.868 ± 1.191	4.291 ± 1.246	P=0.003
UCR (%) P=0.0048				
Q1(≤3.56)	58(24.9%)	6(10.9%)	52(29.2%)	
Q2(3.57-4.31)	58(24.9%)	13(23.6%)	45(25.3%)	
Q3(4.32-5.09)	57(24.5%)	13(23.6%)	44(24.7%)	
Q4(≥5.10)	60(25.7%)	23(41.8%)	37(20.8%)	
PeAF	62(26.61%)	23(41.82%)	39(21.91%)	P=0.004

BMI, body mass index; CAD, coronary artery disease; LAD, left atrial diameter; LVEF, left ventricular ejection fraction; WBC, white blood cells; TG, triglyceride; TC, total cholesterol; LDL-C; low-density lipoprotein cholesterol; HbA1c, glycosylated haemoglobin; BNP, B-type natriuretic peptide; SOD, superoxide dismutase; UA, uric acid; UCR, uric acid:creatinine; PeAF, persistent atrial fibrillation.

The baseline clinical characteristics of patients classified by pre-ablation UCR quartile are listed in **Table 2**. Patients in Q4 had higher UCR (P < 0.001). From the lowest to the highest UCR quartile, participants had increasing levels of UA (P < 0.01), TG (P < 0.01), LDL-C (P < 0.01), WBC (P < 0.05) and neutrophils (P < 0.01). Meanwhile, there were no statistically significant differences in age, sex, BMI, D-dimer, Fib, HbA1c, SOD, LAD, or LVEF among the UCR quartiles.

**Table 2:** Clinical characteristics according to quartiles of UCR level.

	Q1(<3.57) (n=58)	Q2(3.58-4.31) (n=58)	Q3(4.32-5.09) (n=57)	Q4(>5.10) (n=60)	P
Clinical data					
Age(years)	62.43 ± 10.60	63.03 ± 8.21	60.32 ± 8.52	60.00 ± 10.02	P=0.084
Gender, male; n (%)	40(69.0%)	30(51.7%)	30(52.6%)	28(46.7%)	P=0.085
BMI	24.91 ± 3.5	25.6 ± 5.22	26.28 ± 3.34	26.74 ± 3.66	P=0.174
Smoking, n (%)	22(37.9%)	17(29.3%)	13(22.8%)	15(25.0%)	P=0.285
Alcohol, n (%)	25(43.1%)	20(34.5%)	21(36.8%)	17(28.3%)	P=0.411
Hypertension, n (%)	23(39.7%)	29(50.0%)	30(52.6%)	33(55.0%)	P=0.361
Diabetes mellitus, n (%)	13(22.4%)	12(20.7%)	7(12.3%)	8(13.3%)	P=0.361
CAD, n (%)	23(39.7%)	17(29.3%)	20(35.1%)	22(36.7%)	P=0.695
CHA2DS2-vasc Score	2.19 ± 1.88	2.40 ± 1.44	2.35 ± 1.61	2.33 ± 1.64	P=0.916
Echocardiographic parameters					
LAD(mm)	38.40 ± 5.21	39.17 ± 5.45	38.62 ± 5.02	39.13 ± 5.43	P=0.842
LVEF, %	63.04 ± 5.95	63.46 ± 5.39	63.94 ± 5.55	63.38 ± 5.80	P=0.874
Laboratory parameters					
D-dimer, mg/L	0.422 ± 0.460	0.328 ± 0.253	0.314 ± 0.448	0.376 ± 0.571	P=0.586
WBC, ×10^9	6.171 ± 1.819	6.511 ± 1.658	5.659 ± 1.277	5.818 ± 1.335	P=0.018
neutrophil,×10^9	3.771 ± 1.443	4.0 ± 1.553	3.208 ± 1.068	3.27 ± 1.069	P=0.002
lymphocyte,×10^9	1.753 ± 0.530	1.856 ± 0.639	1.863 ± 0.488	1.923 ± 0.564	P=0.430
Fibrinogen, g/L	2.626 ± 0.708	2.589 ± 0.511	2.62 ± 0.654	2.70 ± 0.421	P=0.739
TG, mmol/L	1.068 ± 0.497	1.322 ± 0.635	1.405 ± 0.942	1.515 ± 0.698	P=0.009
TC, mmol/L	3.78 ± 0.917	4.036 ± 0.959	4.108 ± 0.943	4.268 ± 0.953	P=0.056
LDL-C, mmol/L	2.061 ± 0.685	2.297 ± 0.723	2.348 ± 0.689	2.527 ± 0.771	P=0.009
HbA1c,%	6.012 ± 0.853	6.206 ± 0.776	6.067 ± 0.759	6.096 ± 0.710	P=0.641
creatinine, mmol/L	80.66 ± 19.65	74.31 ± 15.20	69.39 ± 11.75	63.66 ± 11.19	P<0.001
UA, mmol/L	242.9 ± 62.39	290.7 ± 62.23	322.7 ± 56.17	381.7 ± 68.60	P<0.001
SOD, U/mL	159.8 ± 17.54	159.0 ± 27.35	161.8 ± 19.89	167.8 ± 15.63	P=0.119
UCR	3.032 ± 0.414	3.909 ± 0.205	4.651 ± 0.190	6.063 ± 1.000	P<0.001

Abbreviations as in **Table 1**.

### 3.2 UCR correlation with AF recurrence after ablation

Univariate Cox regression analysis revealed that UCR (HR 1.299, 95% CI 1.092-1.545, P = 0.003), AF subtype (HR 2.19, 95% CI 1.274-3.763, P = 0.005), TG (HR 1.424, 95% CI 1.064-1.905, P = 0.017) and HbA1c (HR 1.373. 95% CI 1.016-1.856, P = 0.039) were associated with AF recurrence during the follow-up period (**Table 3**). Multivariate Cox regression analysis revealed that UCR (HR 1.217, 95% CI 1.008-1.468, P = 0.041), AF subtype (HR 2.711, 95% CI 1.414-5.199, P = 0.003), and TG (HR 1.437, 95% CI 1.054-1.960, P = 0.022) were associated with AF recurrence. The higher values of UCR were significantly associated with the incidence of AF recurrence. Considering UCR as a categorical variable, after adjustment for age, sex and LAD, there was an increased risk of recurrence in subjects in the highest quartile of UCR compared with subjects in the lowest quartile (HR 4.099, 95% CI 1.636-10.268, P = 0.003).

**Table 3:** Correlation Between UCR and recurrence of AF.

	Model 1		Model 2		Model 3	
	HR(95% CI)	P value	HR(95% CI)	P value	HR(95% CI)	P value
UCR (continuous)	1.299(1.092-1.545)	0.003*	1.271(1.062-1.521)	0.009*	1.217(1.008-1.468)	0.041*
UCR quartiles						
Q1	Reference		Reference		Reference	
Q2	2.117(0.805-5.572)	0.129	2.054(0.777-5.430)	0.147	2.022(0.764-5.352)	0.156
Q3	2.302(0.875-6.057)	0.091	2.222(0.839-5.885)	0.108	2.204(0.832-5.839)	0.112
Q4	4.424(1.800-10.874)	0.001*	4.203(1.684-10.491)	0.002*	4.099(1.636-10.268)	0.003*
AF subtype	2.190(1.274-3.763)	0.005*	2.344(1.359-4.043)	0.002*	2.711(1.414-5.199)	0.003*
TG, mmol/L	1.424(1.064-1.905)	0.017*	1.418(1.051-1.913)	0.022*	1.437(1.054-1.960)	0.022*
HbA1c, %	1.373(1.016-1.856)	0.039*	1.399(1.02-1.917)	0.037*	1.292(0.904-1.847)	0.159
Age	0.989(0.961-1.017)	0.42				
Sex	0.731(0.43-1.241)	0.246				
LAD, mm	1.021(0.97-1.075)	0.433				

Model 1, Unadjusted; Model 2, Adjusted for age,sex; Model 3, Adjusted for age, sex, LAD. Abbreviations as in **Table 1**.

### 3.3 Predictive value of UCR in AF recurrence

**Figure 1:**
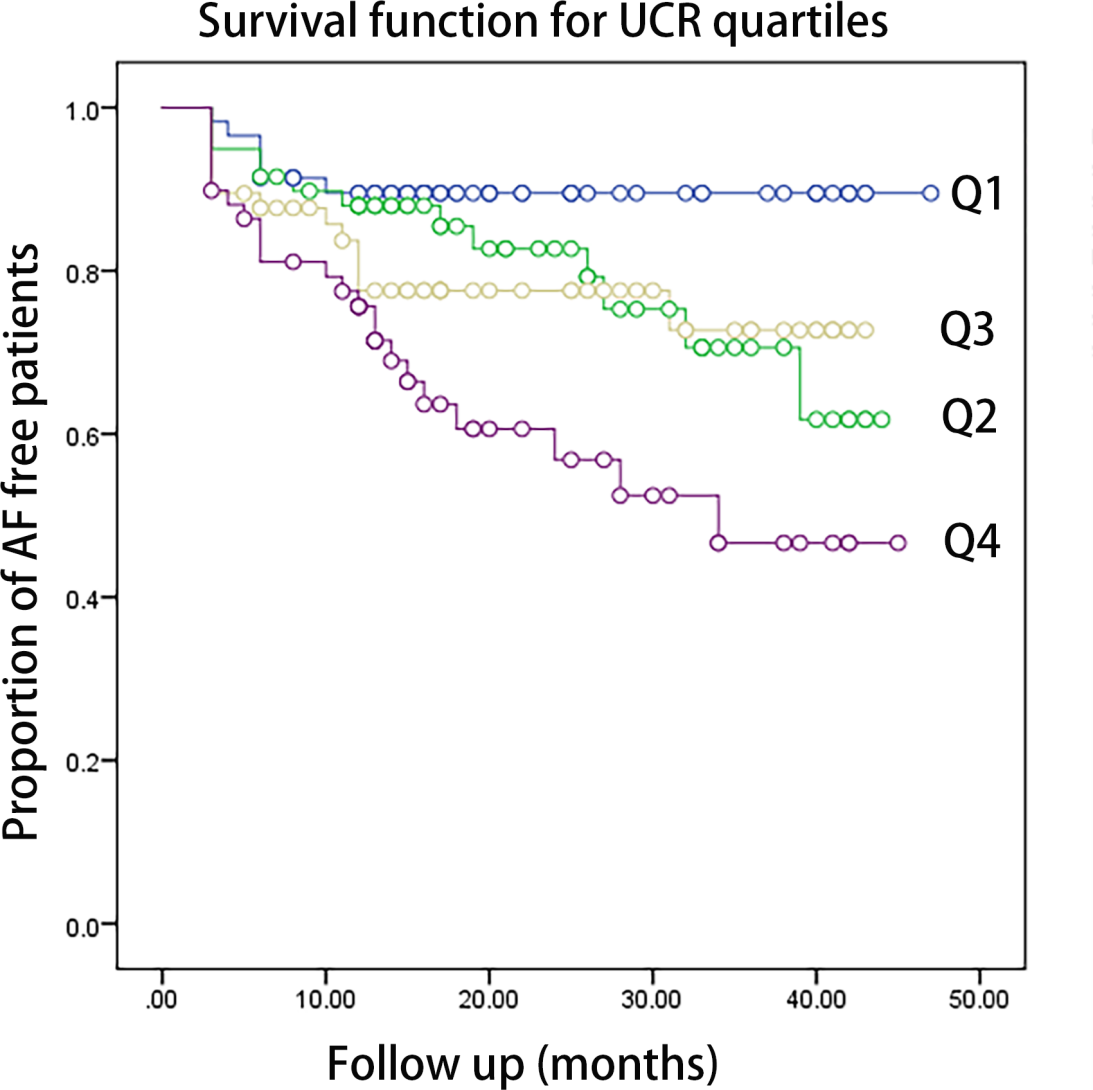
The Kaplan-Meier analysis of the AF free rate during the follow-up period according to UCR quartiles (log-rank P=0.002. Q1: <3.57,Q2: 3.58-4.31, Q3: 4.32-5.09, Q4: >5.10). AF, atrial fibrillation; UCR, uric acid/creatinine ratio.

**Figure 2:**
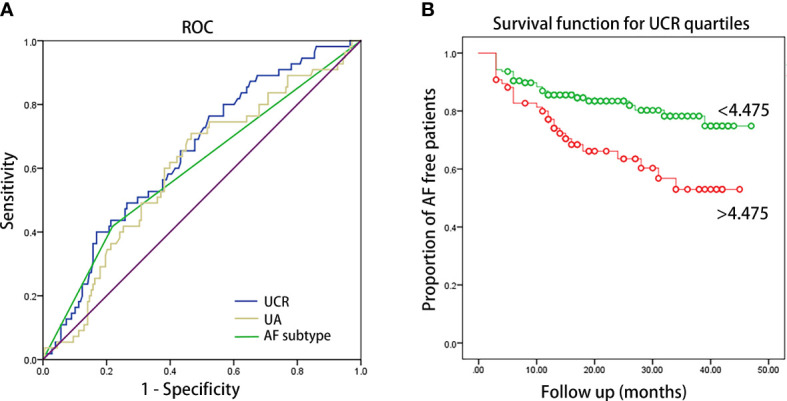
**(A)** ROC curve of UCR, UA and AF subtype for predicting AF recurrence after catheter ablation. The AUC were 0.651 (95% CI: 0.568-0.733, P = 0.001), 0.601 (95% CI: 0.516-0.686, P = 0.024) and 0.59 (95% CI: 0.501-0.680, P = 0.046) for UCR, UA and AF subtype, respectively. AUC: area under the curve. **(B)** Kaplan–Meier survival estimates of AF recurrence in patients with AF undergoing catheter ablation stratified by the pre-ablation UCR level of 4.475.

Kaplan–Meier curves showed time to recurrence of AF in patients within different quartiles of UCR (P = 0.002, **Figure 1**). According to the results of ROC analysis, as shown in **Figure 2**, the area under the curve (AUC) for UCR was 0.651 (95% CI: 0.568-0.733, P = 0.001). UCR exhibited a larger AUC than the SUA (AUC:0.601, 95% CI: 0.516-0.686, P = 0.024) and AF subtype (AUC: 0.59, 95% CI: 0.501-0.680, P = 0.046). A cut-off point of 4.475 for UCR had sensitivity of 65.5% and specificity of 59.6% in predicting AF recurrence (P = 0.001). Kaplan–Meier curves showed time to recurrence of AF in patients with UCR above and below the cut-off level of 4.475 (P = 0.003, **Figure 2**).

### 3.4 UCR in different AF types and genders

Subgroup analysis was conducted according to the different AF type and genders. Patients with PeAF had higher levels of UCR (4.81 ± 1.44 vs. 4.29 ± 1.15, P < 0.001) than PAF. Univariate Cox regression analysis showed that UCR was associated with AF recurrence in PAF (HR 1.426, 95% CI 1.092-1.8608, P = 0.009), but not in PeAF (HR 1.104, 95% CI 0.854-1.426, P = 0.451).

In terms of gender, female patients had higher levels of UCR (4.669 ± 1.375 vs. 4.228 ± 1.113, P < 0.001) than male patients. Univariate Cox regression analysis showed that higher UCR was associated with AF recurrence in male patients (HR 1.407, 95% CI 1.015-1.950, P = 0.04), but there was no significant association in female patients (HR 1.226, 95% CI 0.979-1.535, P = 0.077).

### 3.5 Correlation analysis of UCR with TG, LDL-C and BMI

**Figure 3:**
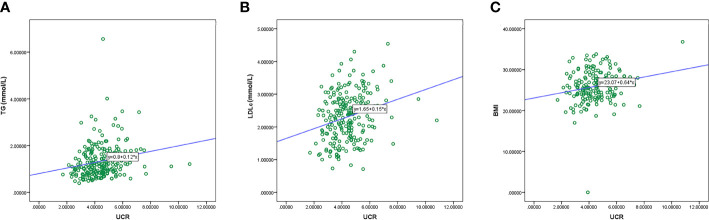
Correlation between the UCR level and duration of TG **(A)**, LDL-C **(B)**, and BMI **(C)**. TG, triglyceride.

Calculation of Spearman’s correlation coefficient showed that there was a positive correlation of pre-procedural UCR with TG (r = 0.276, P < 0.001), LDL-C (r = 0.251, P < 0.001) and BMI (r = 0.160, P = 0.037) (**Figure 3**).

## 4 Discussion

The study revealed that an increase in UCR was positively associated with AF recurrence after radiofrequency catheter ablation. Participants in the highest UCR quartile (Q4) had a significantly elevated risk of AF recurrence than those in the lowest quartile. This increase of UCR in AF recurrence was also statistically significant in PAF and in male patients. Moreover, UCR might be a better predictor of AF recurrence than UA.

AF poses a significant burden on patients, physicians, and healthcare systems globally; however, the effectiveness of therapeutic measures has been unsatisfactory. Catheter ablation of AF appears to be a promising treatment, but recurrence rates are still relatively high. The risk factors influencing the outcome of catheter ablation of AF include not only the type and duration of AF, but also hypertension, obesity, diabetes, hyperlipidaemia, smoking, alcohol consumption, OSA, and physical inactivity. In a retrospective study of 330 patients with paroxysmal AF who underwent catheter ablation, elevated preoperative SUA was associated with a higher rate of recurrence of AF (11). In another prospective study, Canpolat et al. enrolled 363 patients with paroxysmal AF. They demonstrated that SUA level is a powerful and independent predictor of AF recurrence in patients who have undergone successful cryoballoon-based AF ablation (12). Previous studies have shown that, SUA levels were positively correlated with recurrence of AF (11–14). SUA is a metabolic product of purine metabolism. Xanthine oxidase(XO) is a key enzyme in the breakdown of purines and pyrimidines to UA, and is also a critical source of reactive oxygen species (ROS), free radicals responsible for oxidative damage in AF (15). One study found that febuxostat could inhibit atrial electrical and structural remodelling of AF by suppressing XO (16). UA activates NF-KB and MAPK signalling pathways and induces the expression of inflammatory factors and chemokines, which have been connected with AF (17). Inflammation and oxidative stress, both of which promote the progression of the electrical and structural remodelling of AF, also accelerate the recurrence of AF (12).

SUA is increased in acute and chronic renal insufficiency, and renal dysfunction increases the risk of AF recurrence after catheter ablation (18, 19). Recently, renal function-normalised serum UA level has appeared as a new biomarker and is believed to reflect endogenous UA levels more precisely than SUA. Several studies have suggested that serum UCR is significantly associated with chronic obstructive pulmonary disease, chronic kidney disease, and B-Cell function in type 2 diabetes mellitus (20–22). The serum UCR, which represents renal function-normalized SUA, is associated with diverse adverse outcomes. Furthermore, this association was partially mediated through blood lipids, BMI, blood pressure, hs-CRP, and blood glucose (23). In a prospective cohort study, baseline UCR was significantly associated with incident metabolic syndrome (MetS), and UCR may be a better biomarker of incident MetS than SUA by stepwise multiple linear regression analysis, among community-dwelling women (6). A recent longitudinal study on Chinese communities found that renal function-normalised UA was associated with renal disease progression in a cohort of T2DM patients. In addition to predicting metabolism and renal function, UCR also predicts all-cause mortality (8, 24). In the middle age and older population in China, elevated values of UCR were strongly associated with an increased risk of MetS, and this positive relationship remained in those individuals with normal uric acid levels (25). In common with previous studies, the present study showed that UCR is significantly associated with AF recurrence and predicted recurrence with greater sensitivity than UA. This might be because UCR is a global index of SUA and creatinine metabolism, so that it is a better indicator than a single index. In our study, in addition to pre-ablation UCR level, we also found that AF subtype, TG, HbA1c, and UA levels were independent predictors of AF recurrence. Correlation analysis showed that there was a positive correlation of pre-procedural UCR with TG, LDL-C and BMI. In a chinese study, serum UCR are strongly associated with the risk of MetS in postmenopausal Chinese women (26). In addition, a significant increase was observed in the prevalence of overweight/obesity, hypertension, and dyslipidemia across the SUA quartiles in Patients With Type 2 Diabetes Mellitus (27).

The study results corroborated those of previous studies and strengthened the relationship of SUA and creatinine in AF recurrence after catheter ablation. In this study, LAD was not found to be a predictor of AF recurrence. A possible reason is that the LA of the population we included had relatively small diameter. This is one of the reasons for the low AF recurrence rate in this study. In subgroup analysis, this study suggests that UCR is a valuable predictive biomarker in AF recurrence in patients with PAF and in male patients. In female patients and those with PeAF, UCR has no predictive value for AF recurrence. We speculated on the possible reasons. Several studies also reported that there are sex-related differences in clinical characteristics in the section of the AF population who are free of valvular disease. Women with AF are older than their male counterpart, and have a higher heart rate, more symptoms, more complications, a poorer quality of life, worse prognosis, and increased prevalence of PeAF, stroke and death (28–30). Our data showed a tendency for female patients to have an increased risk of recurrence after catheter ablation, which was in agreement with previous findings. In addition, the reason for the finding that UCR level does not predict recurrence of PeAF is elusive. It is widely known that the recurrence rate in patients with PeAF is significantly higher than that in patients with PAF. The reasons may be the longer duration of episodes of AF, larger atrial volume and severe atrial fibrosis in PeAF, which are the more important risks for the recurrence of PeAF.

Our study had several limitations. First, the diagnosis of AF recurrence was based on the occurrence of palpitations symptoms, periodic phone calls, ECG and Holter recordings. But ECG and Holter were done every three or six months, it might not catch the rhythm of a patient with PAF, or it might not be done in time for an AF attack. Therefore, we underestimated the true incidence of AF relapse in our study possibly. Second, we did not measure other markers of specific oxidative stress and inflammation, such as IL-6, TNF-α and ROS. Finally, this study was a retrospective study performed with data from a single centre with a relatively small sample size and short follow-up period. Therefore, further prospective studies with a larger number of patients and longer follow-up period may be needed to confirm and enhance our results.

## 5 Conclusion

UCR was significantly associated with AF recurrence after catheter ablation. However, further studies are required to identify the appropriate parameters of SUA or MetS for predicting recurrence of AF.

## Data availability statement

The raw data supporting the conclusions of this article will be made available by the authors, without undue reservation.

## Ethics statement

The studies involving human participants were reviewed and approved by The First Affiliated Hospital of Shandong First Medical University. The patients/participants provided their written informed consent to participate in this study.

## Author contributions

YZ was responsible for data collection, statistical analysis and paper writing. YW was responsible for data collection and follow-up. XY was responsible for the follow-up of some patients. ZL was responsible for informed consent signing and preoperative data collection. LS is responsible for the writing and guidance of the thesis. YH is responsible for the project design and paper revision. All authors contributed to the article and approved the submitted version.
